# Comparison of malignancy and spatial distribution between latent and clinical prostate cancer: an 8-year biopsy study

**DOI:** 10.1186/s40001-022-00801-0

**Published:** 2022-09-10

**Authors:** Liang Zhen, Zhou Zhien, Huang Hanzi, Wu Xingcheng, Xiao Yu, Wang Wenze, Zuo Yuzhi, Chen Yuliang, Zhou Yi, Yan Weigang

**Affiliations:** 1 Department of Urology, Peking Union Medical College Hospital, Peking Union Medical College, Chinese Academy of Medical Sciences, No.1 Shuaifuyuan, Wangfujing, Dong Cheng District, Beijing, 100730 China; 2 Department of Pathology, Peking Union Medical College Hospital, Peking Union Medical College, Chinese Academy of Medical Sciences, No.1 Shuaifuyuan, Wangfujing, Dong Cheng District, Beijing, 100730 China

**Keywords:** Autopsy, Latent cancer, Prostate cancer, Spatial distribution, Malignancy

## Abstract

**Background:**

Current prostate cancer (PCa) screening may detect nonprogressive lesion, leading to overdiagnosis and overtreatment. The purpose of the present study is to investigate whether the tumor pathological origin of latent prostate cancer (lPCa) and clinical prostate cancer (cPCa) are consistent, and to verify the current clinically significant prostate cancer criteria.

**Methods:**

Prostate specimens were obtained from postmortem autopsy between 2014 and 2021 and patients who went through radical prostatectomy from 2013 to 2021. The pathological characteristics and spatial distribution of the lPCa group and cPCa group were compared and analyzed through SPSS software with *P* < 0.05 representing statistical significant.

**Results:**

In lPCa group, a total of 45 tumor lesions from 24 lPCa cases were included, 54.2% of lPCa patients were ISUP ≥ 2, 12.5% had tumor volume ≥ 0.5 ml, and 16.7% had extraprostatic extension (EPE). In cPCa group, there were a total of 429 tumor lesions in 126 cases, 92.1% of cPCa patients were ISUP ≥ 2, and 82.5% had tumor volume of ≥ 0.5 ml. 36.3% had EPE. LPCa and cPCa have the same spatial distribution characteristics, and no significant difference was detected between the anterior and posterior zone. Peripheral zone tumors were significantly more common than transitional zone tumors. Tumors in apical 1/3 and middle 1/3 were significantly more common than basal 1/3.

**Conclusion:**

The malignancy of cPCa is significantly higher than that of lPCa, and the spatial distribution of cPCa and lPCa is consistent. ISUP grade 2 is not sufficient to determine clinical significance of tumor.

## Introduction

Prostate cancer (PCa) is the second most frequent urological malignancies in men globally [1]. Clinical prostate cancer (cPCa) is defined as PCa that leads to prostate cancer-related symptoms or death. The traditional screening method for PCa contains digital rectal examination (DRE) and evaluation of prostate-specific antigen (PSA) level [2]. PSA, as the most recognized and most commonly applied biomarker of PCa in clinic, plays a significant role in diagnosing PCa. However, there remain uncertain scenarios because PSA is secreted by both PCa cells and normal/benign hyperplastic prostatic epithelia. Serum PSA levels could be greatly affected by the prostate volume, leading to a high rate of unnecessary biopsies and over detection of indolent PCa [3]. The pathological definition of clinically significant prostate cancer (csPCa) included three criteria: index tumor volume (ITV) > 0.2 cm^3^, Gleason score > 7, or extracapsular extension (EPE) [4]. Generally, lesions that meet abovementioned criterion intend to adversely affect the health of patients [5]. Many indolent prostate cancers remain asymptomatic and undiagnosed throughout lifetime [6], and those first detected by autopsy are known as latent prostate cancer (lPCa). Nevertheless, in a meta-study with 18 researches, Jacklin et al. reported that the Gleason score of 25% lPCa cases were > 7 and the average volume of the cancer lesion was 0.516 ml [7]. Since the original definition of pathologically csPCa was proposed more than 20 years ago, it is necessary to evolve the current standard.

Up to now, most studies on the spatial distribution of the prostate cancer are according to the samples confirmed by radical prostatectomy (RP) or prostate biopsy. Limited by the difficulty in obtaining specimens, the quantity of autopsy studies on PCa in the worldwide is relatively low, and the majority of them are performed in North America, Europe, and Japan. At present, there is no comparative study on the pathological characteristics of lPCa and cPCa in China. Therefore, the present study aims to describe the characteristics and spatial distribution of PCa through comparing the pathological characteristics of lPCa specimens from autopsy and cPCa specimens from RP. We hope to explore the tumor predilection site of PCa and evaluate the clinical significance of current csPCa standards.

## Materials and methods

### Patients enrollment

All specimens in the lPCa group were obtained from the Human Tissue Organ Bank of the Department of Anatomy and Histo-Embryology, Peking Union Medical College from 2014 to 2021. Only specimens with intact prostate glands, capsules, seminal vesicles, and pathologically diagnosed with PCa after death were included; specimens from donors who had been definitively diagnosed with PCa or died of PCa were excluded.

All specimens in cPCa group were obtained from RP in the Department of Urology, Peking Union Medical College Hospital from November 2013 to August 2021. All patients have given written informed consent to publication of their case details. Patients with regional lymph node and distant metastasis proved by preoperative imaging examinations were excluded. Patient who received neoadjuvant androgen deprivation therapy or previous prostate-related surgery were also excluded.

Our research has been reviewed and approved by the Ethics Committee of the Basic Research Laboratory of the Chinese Academy of Medical Science.

### Encapsulation of whole prostate specimen

**Fig. 1:**
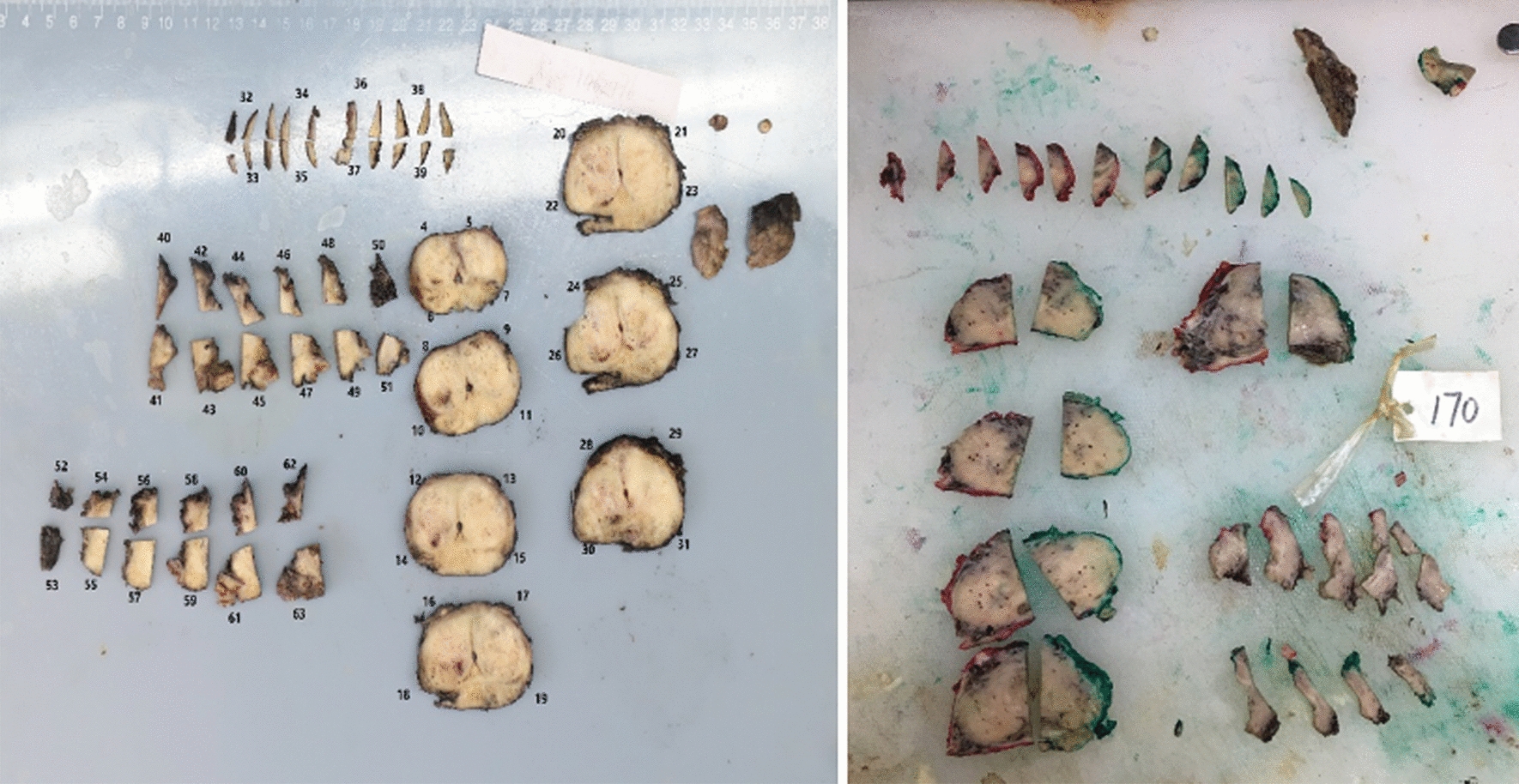
Segmentation of whole prostate specimen. The distal 5 mm (apical) were coned and sagittally sliced with an interval of 2 mm. The rest of the gland was transversely sliced from distal to proximal with an interval of 3 mm (middle), leaving the proximal 5–10 mm (basal) of the prostate to be sagittally sliced with an interval of 2 mm

The prostate specimens were sliced perpendicularly to the long axis (Fig. 1). All specimens were immersed in 10% formalin solution for fixation and embedded in paraffin for HE staining. Pathological reading was completed through NDP View 2.0 software (Hamamatsu Photonics K.K, Japan). Pathological diagnoses of all prostate specimens were performed by a urological pathologist with more than 20 years of experience.

**Fig. 2:**
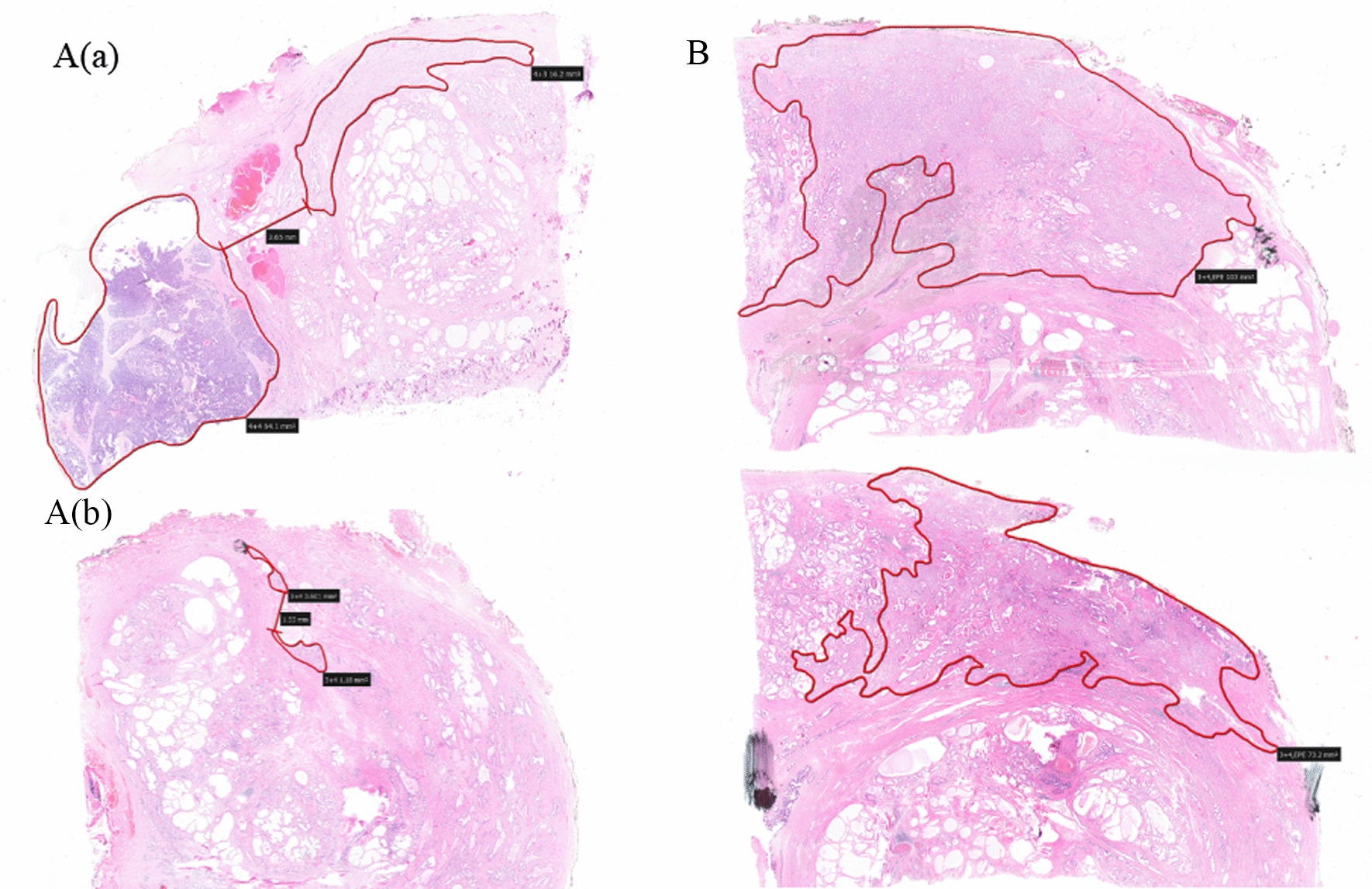
Prostate tissue digital section. **A**: **a** The area delineated by the red line is a prostate cancer lesion; the distance between the edges of the two cancer lesion is > 3 mm, which means they do not belong to the same tumor; **b** The area delineated by the red line is a prostate cancer lesion, and the distance between the two edges is < 3 mm, which means they belongs to the same tumor. **B**: **a** and **b** are the digital slices of the prostate tissue at adjacent layers in the vertical direction. The area delineated by the red line is the prostate cancer lesion. The projections of the cancer lesion on the two layers belong to the same tumor

The pathological scoring of PCa was based on the International Society of Urological Pathology (ISUP) classification. The outline of the cancer lesion was delineated layer by layer along the boundary between the tumor and the normal tissue. The total volume of each tumor was calculated through the following formula: tumor volume = tissue shrinkage coefficient × sum of tumor area at each layer × layer thickness. The tissue shrinkage coefficient of cPCa and lPCa is 1.12 [8] and 1.5, respectively [9] (Fig. 2). ITV was defined as the tumor with the highest Gleason score. The total prostate volume (PV) was calculated from the recorded specimen dimensions (length, width, and height) using the ellipsoid formula PV = height × width × length × π/6. For more accurate calculation of prostate volume, the whole mount prostate specimens for both groups were separated from the seminal vesicles and periprostatic fat [10, 11].

In the horizontal direction, prostate was divided into anterior and posterior zones bounded by the coronal plane of the urethra and divided into peripheral zone (PZ) and transitional zone (TZ) according to the tissue morphology. Furthermore, vertically, the prostate was divided into three parts, which were defined as apical 1/3, middle 1/3, and basal 1/3, respectively, from distal to proximal.

All data analysis was performed using SPSS software (version 26.0, IBM SPSS Inc., Armonk, NY, USA). If the data followed normal distribution, it is represented as mean values ± standard deviation. Otherwise, the data were described through median with interquartile range (IQR). Independent sample *T* and Mann–Whitney tests were applied for the comparison of parametric and nonparametric continuous data, respectively. All tests were two sided, and *P* < 0.05 was considered statistically significant. According to previous studies, men older than 70 years were more likely to be diagnosed with larger and higher Gleason score PCa than younger men [12]; thus, in the present study, age matching was performed for each group to avoid the influence of age difference.

## Results

### Basic characteristics of the enrolled patients

In this study, a total of 24 and 126 specimens were included in the lPCa and cPCa group separately. The median (IQR) age for lPCa group was 74 (53–80) years old; and the median (IQR) age for cPCa group was 71 (52–80) years old. There was no significant difference in median age between the two groups after age matching. Patient characteristics are displayed in Table 1.

**Table 1:** Comparison of malignancy between 1PCa group and cPCa group

Characteristics	Latent prostate cancer	Clinical prostate cancer	*P* value	OR (95% CI)
Total patient number	24	126	–	–
Total lesion number	45	429	–	–
Median age (IQR)/ (%)	74 (64–77)	71 (63–74)	0.149	0.626 (0.698–1.174)
ISUP grade ≥ 2	13	116	< 0.001	10.39 (3.64–29.69)
Tumor volume ≥ 0.5 ml	3	104	< 0.001	32.68 (8.95–119.36)
EPE	4	46	< 0.001	6.54 (2.11–20.33)
csPCa	13	119	< 0.001	14.65 (4.80–44.72)

According to Table 1, compared with lPCa, the tumor malignancy was significantly higher in cPCa group, cPCa had a significantly higher probability of having ISUP grade ≥ 2, tumor volume ≥ 0.5 ml or EPE, with the odds ratio (OR) = 10.39 (95% confidence interval [CI]: 3.64–29.69 *P* < 0.001), 32.68 (95%CI: 8.95–119.36 *P* < 0.001), and 6.54 (95%CI: 2.11–20.33 *P* < 0.001), respectively. The quantity of clinically significant tumors in cPCa group was also significantly higher than that in lPCa group (*P* < 0.001).

### Tumor malignancy of latent prostate cancer and clinical prostate cancer

The basic characteristics of lPCa are shown in Table 2. In lPCa group, there were a total of 45 tumor lesions from 24 lPCa cases. The median prostate volume and tumor volume were 29.4 ml and 0.009 ml, respectively, with median ITV = 0.032 ml. As for the csPCa analysis based on index tumor lesion, 3 cases were ≥ 0.5 ml, accounting for 12.5% of the total. 54.2% of the lPCa cases had ISUP grade ≥ 2, and 11 (45.8%), 8 (33.3%), 0 (0%), 1 (4.2%), and 4 (16.7%) were ISUP grades 1–5, respectively, and 16.7% patients had EPE. Concurrently, in total 45 tumor lesions, 21 of them were ISUP grade ≥ 2, and among which 9.5% were ≥ 0.5 ml, 23.8% had EPE. Among 16 ISUP grade ≥ 2 tumor lesions meeting the condition of tumor volume < 0.5 ml and confined to the prostate, 12 of them (75.0%) were ISUP grade 2.

**Table 2:** Basic characteristics of lPCa and cPCa patients

Characteristics median (IQR)/ (%)	lPCa	cPCa
Prostate volume	29.4 (27.5–40.2)	34.5 (27.8–44.7)
Total tumor volume/ml	0.055 (0.007–0.304)	2.128 (0.822–4.566)
Index tumor volume/ml	0.032 (0.004–0.138)	1.545 (0.466–4.045)
Tumor volume group		
Volume ≥ 0.5 ml	3 (12.5)	104 (82.5)
Volume < 0.5 ml	21 (87.5)	22 (17.5)
ISUP classification		
1	11 (45.8)	10 (7.9)
2	8 (33.3)	59 (46.8)
3	0 (0.0)	33 (26.2)
4	1 (4.2)	10 (7.9)
5	4 (16.7)	14 (11.1)
EPE	4 (16.7)	46 (36.3)
csPCa	13 (54.2)	119 (94.4)
Spatial distribution of tumors		
Anterior zone	12 (50.0)	63 (50.0)
Posterior zone	10 (41.7)	50 (39.7)
Both anterior and posterior zone	2 (8.3)	13 (10.3)
Peripheral zone	19 (79.2)	82 (65.1)
Transitional zone	4 (16.7)	15 (11.9)
Both peripheral and transitional zone	1 (4.2)	29 (23.0)
Apical 1/3 region	12 (50.0)	111 (88.1)
Middle 1/3 region	16 (66.7)	111 (88.1)
Basal 1/3 region	3 (12.5)	50 (39.6)

The basic characteristics of cPCa are shown in Table 2. In cPCa group, there were a total of 429 tumor lesions in 126 cases with the median prostate volume = 34.5 ml. The median tumor volume was 0.044 ml (0.006–0.461 ml), with the median ITV = 1.545 ml. As for the csPCa analysis according to index tumor lesion, the tumor volumes of 104 cases were ≥ 0.5 ml, accounting for 82.5% of the total. The ISUP grade of 92.1% cases were ≥ 2, and 10 (7.9%), 59 (46.8%), 33 (26.2%), 10 (7.9%), and 14 (11.1%) were ISUP grades 1–5, respectively, and 36.3% patients had EPE. In total 429 tumor lesions, 359 of them were ISUP grade ≥ 2, and among which 28.1% were ≥ 0.5 ml, 22.6% had EPE. Among 242 ISUP grade ≥ 2 tumor lesions meeting the condition of tumor volume < 0.5 ml and confined to the prostate, 205 of them (84.7%) were ISUP grade 2.

### Tumor spatial distribution of latent prostate cancer and clinical prostate cancer

As shown in Table 3, the intra-group analysis demonstrated that the index tumors were more likely to occur in PZ than TZ, and the quantity of tumors involving the apical 1/3 and middle 1/3 was significantly higher than that of the basal 1/3 for both lPCa and cPCa groups (*P* = 0.040). However, there was no statistical difference in the distribution of lesions between the anterior and posterior zones.

**Table 3:** Spatial distribution characteristics of lPCa group and cPCa group

Characteristics	Latent prostate cancer		Clinical prostate cancer		OR (95%CI)	*P* (lPCa vs cPCa)
Index tumor lesion						
Anterior	12	*P* = 0.670	63	*P* = 0.221	0.993 (0.393–2.508)	*P* = 0.989
Posterior	10		50			
Both anterior and posterior	2		13		–	
Peripheral zone	19	*P* = 0.002	82	*P* < 0.001	0.774 (0.221–2.707)	*P* = 0.688
Transitional zone	4		15			
Both peripheral zone transitional zone	1		29		–	
Apical 1/3	12	*P* = 0.040	111	*P* = 0.042	–	
Middle 1/3	16		111		0.749 (0.339–1.656)	*P* = 0.475
Basal 1/3	5		84		1.819 (0.617–5.365)	*P* = 0.278
Total tumor lesion						
Anterior	23	*P* = 0.435	206	*P* = 0.843	1.247 (0.653–2.382)	*P* = 0.504
Posterior	18		202			
Both anterior and posterior	4		21		-	
Peripheral zone	36	*P* < 0.001	350	*P* < 0.001	0.754 (0.316–1.798)	*P* = 0.524
Transitional zone	7		50			
Both peripheral zone transitional zone	2		29		–	
Apical 1/3	22	P = 0.004	285	*P* = 0.004		
Middle 1/3	28		234		0.655 (0.364–1.177)	*P* = 0.157
Basal 1/3	8		165		1.616 (0.702–3.717)	*P* = 0.259

As for the total tumor aspect, intra-group analysis verified that tumor lesions were more prone to occur in PZ than TZ, and no significant difference was detected in tumor distribution between the anterior and posterior zones for both two groups. As for the vertical direction, in lPCa group, the tumors involving basal 1/3 were significantly less than those involving apical 1/3 and middle 1/3. Moreover, in cPCa group, tumor lesions were more likely to occur at the apical 1/3 when compared with the middle 1/3 (*P* = 0.025), while that difference was not detected in lPCa group (*P* = 0.396).

In addition, we further compared the spatial distribution of tumor lesions between lPCa and cPCa. Our results in Table 3 indicated that for both index and total tumor lesion, there was no significant difference in spatial distribution between the two groups in horizontal direction (anterior and posterior), vertical direction (apical 1/3 middle 1/3 and basal 1/3), and morphology aspect (peripheral and transitional zone). The spatial distribution of the two groups has the same characteristics: the majority of lesions are located at PZ, with roughly equal distribution in the anterior and posterior zones. Lesions located at the apical 1/3 and middle 1/3 are more common than those located at the basal 1/3.

## Discussion

This study is, to the best of our knowledge, the first to explore the difference between the pathological malignancy and the origin of prostate cancer by comparing autopsy findings and clinical specimens. Although lPCa does not cause any clinical symptoms and affect patient survival, some of them are still classified as csPCa according to Epstein criteria. Based on previous study, clinically significant lPCa accounts for about 45–51% of the total in Japanese, and 30–46% in European and American population [12–15]. All these aforementioned results suggested that current Epstein criteria needed to be revised [14]. In the present study, 54.2% (13/24) of lPCa were clinically significant, which is similar to previous autopsy studies. Furthermore, our study also found that in lPCa group, 7 (53.8%) patients were classified as csPCa only due to ISUP = 2. While in cPCa group, 11 (9.2%) of the patients were assigned into csPCa group only due to ISUP = 2. This led us to consider whether ISUP = 2 is appropriate as a determinant for csPCa. Nevertheless, narrowing the criteria for csPCa would lead to an increased risk of missed diagnosis. How to strike a balance between over- and under-diagnosis in clinical practice would be an issue that needs urgent attention in the future.

The comparison of the tumor spatial distribution of lPCa and cPCa could shine a light on pathological origin of PCa and help us better investigate the difference in predilection sites between latent and clinical cancers. In 1977, Breslow et al. conducted an autopsy study on a population of multiple ethnic groups from 7 regions of the world, and the results suggested that tumors distributed in the anterior and posterior zone of the prostate were roughly the same [16]. In the present study, we also detected no significant difference in the distribution of PCa between the anterior and posterior zone in both cPCa group and lPCa group, which means lesions from the aforementioned zones have the same pathological origin. Nevertheless, some previous international studies have offered different point of views that tumors located at the anterior prostate region only account for about 10–30% of total PCa lesions [17–19]. The variation could be explained by the different biopsy approach. In our research, the approach we applied is transperineal prostate biopsy, which is more likely to diagnose tumors located at anterior zone than traditional transrectal biopsy. According to the study of Hossack et al., different prostate biopsy approaches may have an impact on the tumor detection rate of different prostate regions, and transperineal prostate biopsy performs better to detect tumors in anterior zone [20].

Breslow et al. also demonstrated that as the tumor size of lPCa increased, the tumor distribution intended to spread from PZ to TZ, and in general, most latent lesions were distributed in PZ [21]. However, the proportion of tumors in TZ observed in different autopsy studies fluctuated greatly [22–24]. Zlotta et al. indicated that Asian populations have a larger proportion of TZ tumors than European and American populations [15]. In the present study, 79.2% (19/24) of lPCa were distributed in PZ, and 16.7% (4/24) were distributed in TZ with a significant difference. As for cPCa, in 1988 McNeal et al. performed pathological analysis on specimens from RP and found that about 70% of prostate cancers were distributed in PZ and only about 10–20% are distributed in TZ [21]. Since then, multiple studies have verified this conclusion [25–28]. In the present study, 82 (65.1%) of the 126 cPCa lesions were located at PZ, and 15 (11.9%) were located at TZ which also met the results of former study.

In Breslow et al.’s study, they pointed out that the proportion of lPCa lesion located at the apex of the prostate is significantly higher than the basal [16, 29]. In 2002, Takashima found that cPCa is often detected at apex (82.3%) and middle (85.5%) of the prostate, which is significantly more common than basal region according to their RP specimens [30]. Our autopsy results demonstrated that tumors involving the apical 1/3 and the middle 1/3 were significantly more common than those involving the basal 1/3, which is consistent with previous study. Similarly, as for the cPCa, the tumor detection rates of the apical and middle 1/3 were also significantly higher than that of basal 1/3.

This study has several notable limitations. First of all, this was a single-center study with a small sample size. Our study was limited by the different sample sizes between the two groups (24 vs 126); nevertheless, we gave particular attention to Levene’s test for homogeneity in order to test the differences between population variances, but they did not differ significantly (*P* = 0.262). In addition, all lPCa samples were obtained from body donations which would lead to an older age compared with former study. Thus, age matching for two groups were conducted to reduce the confounding effect of age. Secondly, we defined the area with more than 2/3 volume distribution of the tumor as the main distribution area. However, due to the destruction of normal tissue structure by cancer lesion, there may exist some deviation from the actual situation in distinguishing the PZ and TZ from histological morphology. Thirdly, different time intervals from death to autopsy may affect the accuracy of histological and pathological diagnosis under microscope. We expect that future study could explore the differences between lPCa and normal prostate tissue, lPCa and cPCa from the molecular biology perspective, and eventually establish a gene mutation map to help the clinical more accurately diagnose PCa that affects the prognosis of patients.

## Conclusion

The malignancy of cPCa is much higher than lPCa. ISUP grade 2 is not an appropriate determinant for classifying csPCa. Furthermore, there is no significant difference in the distribution of tumors in anterior and posterior zone between lPCa and cPCa. The tumors in PZ are significantly more common than those in TZ. Tumors in apical 1/3 and middle 1/3 are significantly more common than those involving the basal 1/3. There is no significant difference in tumor spatial distribution between lPCa and cPCa. LPCa could be considered as an early stage cPCa without any clinical symptoms.

## Data Availability

Individual study data that underlie the results reported in this article can be made available to the scientific community after de‐identification and upon submission of a data request application to the investigator board via the corresponding author.

## Abbreviations

CI: Confidence interval
cPCa: Clinical prostate cancer
csPCa: Clinically significant prostate cancer
EPE: Extraprostatic extension
ISUP: International Society of Urological Pathology
IQR: Interquartile range
ITV: Index tumor volume
lPCa: Latent prostate cancer
OR: Odds ratio
PCa: Prostate cancer
PZ: Peripheral zone
RP: Radical prostatectomy
TZ: Transitional zone
